# MiR-202-3p determines embryo viability during mid-blastula transition

**DOI:** 10.3389/fcell.2022.897826

**Published:** 2022-08-08

**Authors:** Ruiqin Hu, Yanna Xu, Bingshe Han, Yi Chen, Wenhao Li, Guijun Guan, Peng Hu, Yan Zhou, Qianghua Xu, Liangbiao Chen

**Affiliations:** ^1^ International Joint Research Centre for Marine Biosciences (Ministry of Science and Technology), College of Fisheries and Life Science, Shanghai Ocean University, Shanghai, China; ^2^ Key Laboratory of Exploration and Utilization of Aquatic Genetic Resources (Ministry of Education) and International Research Centre for Marine Biosciences, College of Fisheries and Life Science, Shanghai Ocean University, Shanghai, China; ^3^ Key Laboratory of Sustainable Exploitation of Oceanic Fisheries Resources, College of Marine Science, Shanghai Ocean University, Shanghai, China

**Keywords:** miR-202-3p, mid-blastula transition, NF-κB, apoptosis, zebrafish

## Abstract

Developmental growth is an intricate process involving the coordinated regulation of the expression of various genes, and microRNAs (miRNAs) play crucial roles in diverse processes throughout animal development. The mid-blastula transition (MBT) is a developmental milestone when maternal RNAs are cleared and the zygotic genome programmed asynchronous cell division begins to drive embryogenesis. While mechanisms underlying MBT have been intensively revealed, factors regulating cell proliferation at the transition remain largely unknown. We report here a microRNA, miR-202-3p to be a key factor that determines embryonic fate during MBT in zebrafish. A miR-202-3p antagomir specifically terminated embryo development at the mid-blastula stage. *In vivo* deletion of the miR-202 locus recapitulated the fatal phenotypes, which were rescued only by miR-202-3p or its precursor. Transcriptome comparison revealed >250 RNAs including both maternal and zygotic origins were dysregulated at MBT in the miR-202^−/−^ embryos, corresponding with arrays of homeostatic disorders leading to massive apoptosis. A trio of genes: *nfkbiaa*, *perp* and *mgll*, known to be intimately involved with cell proliferation and survival, were identified as direct targets of miR-202-3p. Importantly, over- or under-expression of any of the trio led to developmental delay or termination at the blastula or gastrula stages. Furthermore, *nfkbiaa* and *perp* were shown to inter-regulate each other. Thus, miR-202-3p mediates a regulatory network whose components interact closely during MBT to determine embryonic viability and development.

## Introduction

The Maternal-to-Zygotic Transition (MZT) is a period in which control of embryo development transitions from reliance on maternally supplied factors to newly synthesized zygotic gene products (Schier 2007). During MZT, two interacting processes are undertaken: the maternal RNAs are cleared and the zygotic genome is activated (ZGA) (Newport et al., 1982; Tadros et al., 2009). MZT occurs in all animals but at different timing depending on the animal species. In zebrafish, mid-blastula transition (MBT) phase is occurs at the 10th cell cycle, which corresponds to the timing of the MZT at approximately 3.5 h post fertilization (hpf) (Kimmel et al., 1995). It also marks the commencement of asynchronous cell division, apoptosis, appearance of cell cycle checkpoints and cell motility, in contrast with the synchronous cleavage divisions in the earlier cell cycles when development is controlled by maternally stored factors (Langley et al., 2014). Especially, the S-phase lengthens, gap phases appear, and cell cycles become sensitive to DNA damage (Jukam et al., 2017). These changes are prerequisite for acquiring different cell fates and specific morphological forms during development (Siefert et al., 2015).

The factors regulating maternal RNA clearance and ZGA have been intensively investigated (Lee et al., 2014; Zhang et al., 2017). A few conserved zygotic microRNAs, such as miR-430 in zebrafish (Giraldez et al., 2006), miR-427 in frog (Lund et al., 2009), and miR-290 in mouse (Tang et al., 2007), play a role in maternal RNA degradation via post-transcriptional regulation in vertebrates (Svoboda et al., 2010; Yartseva et al., 2015). The dramatic changes that occur at the onset of MBT in aquatic animal species are precisely timed in the embryo. Cellular and molecular mechanisms such as nucleus-cytoplasm ratio (Schulz et al., 2019), chromatin architecture remodelling (Hug et al., 2017), DNA methylation patterns (Liu et al., 2018), concentrations of nuclear histones (Joseph et al., 2017; Pálfy et al., 2017) and cytoplasmic polyadenylation-mediated translational control of maternal mRNAs (Winata et al., 2018; Schulz et al., 2019) have been shown to underlie ZGA. Transcription factors such as Zelda, *nanog*, *pou5f3* and *soxB1* are identified to implicate in ZGA (Liang et al., 2008; Lee et al., 2014; Veil et al., 2019). Although much has been learned regarding MZT, it has been estimated that both miR-430s and Ythdf2 pathways still account for only a portion of total maternal mRNA clearance (Giraldez et al., 2006; Zhao et al., 2017).

MicroRNAs are a class of small non-coding RNAs approximately 22 nucleotides that performs significant role in controlling the messenger RNA (mRNA) stability and translation at cellular levels. MiRNAs play essential roles in a wide variety of biological processes, including cellular differentiation, growth, development and metabolism pathways (Bhattacharya et al., 2017). Dicer is required for mature miRNA biogenesis and loss of Dicer would inhibit production of all Dicer-dependent miRNAs. The MZdicer (maternal and zygotic dicer mutant) larvae showed retarded growth and died 2 weeks after fertilization, suggesting that miRNA functions are essential for post-embryonic development (Giraldez et al., 2005). The zebrafish miR-430 family is the most abundant miRNA family during early embryogenesis and is the first expressed during MZT (2.75 hpf). Surprisingly, miR-430 duplex rescued most of the MZdicer embryos as observed during the first 24 h that gastrulation defect (Giraldez et al., 2005). Therefore, miR-430 is an essential miRNA during zebrafish development with striking impacts on morphogenesis (Giraldez et al., 2006). Many other microRNAs like miR-30a are also important for zebrafish embryonic development (O'Brien et al., 2014). In mice, disruption of the miR-137 primary transcript results in early embryonic lethality (Crowley et al., 2015). miR-15 and miR-16 are involved in *Xenopus laevis* embryonic development through the regulation of the Wnt and Nodal signaling pathways (Shi et al., 2009). In *C. elegans*, lin 4 and let 7 were identified as controllers of the timing of larval development: mutations of these genes resulted in the reiteration of larval cell fates and retarded the final differentiation of subsets of specialized cells (Lee et al., 1993; Reinhart et al., 2000).

The miR-202 locus produces two types of mature microRNAs, miR-202-3p and miR-202-5p, are specific and highly conserved in vertebrates (Dai et al., 2009). MiR-202 functions to maintain spermatogonial stem cells in mouse (Chen et al., 2017; Chen et al., 2021). miR-202-3p controls the proliferation, apoptosis, and synthesis function of human sertoli cells (Yang et al., 2019). The reduction of miR-202-5p expression in medaka (*Oryzias latipes*) resulted in impairments of the early steps of oogenesis/folliculogenesis, leading to dramatically reduced female fecundity (Gay et al., 2018). However, maternal loss of miR-202-5p impaired PGC migration in zebrafish (Jin et al., 2020). Acting as a tumor suppressor, dysregulation of miR-202-3p is found to be associated with numerous cancerous transformations (Zhang et al., 2020; Wang et al., 2021).

In the study of the functions of the miR-202 locus in zebrafish development, we occasionally found that injection of a miR-202-3p antagomir into the fertilized eggs specifically terminates cell proliferation and embryogenesis at approximately 4 hpf, with no embryo progression to the epibolic stages. The blastomere cells stop proliferation but enter apoptosis instead. We then deleted the miR-202 locus from the zebrafish genome and carried out in-depth studies on the developmental consequences of this mutation and the underlying mechanisms of the cellular effects resulting from loss of miR-202-3p. We report here that miR-202-3p is a factor involved in ZGA regulation, in which it forms an inter-regulated network with its target genes in the NFκB and P53 related signaling pathways required for preventing cells entering apoptosis. The miR-202-3p-meidated regulatory network thus links ZGA with cell proliferation and survival during mid-blastula transition.

## Material and methods

### Fish

Zebrafish (*D. rerio*, AB strain) were maintained at 28.5°C under standard conditions. Embryos were raised and maintained at 28.5°C and staged according to standard morphological criteria (Kimmel et al., 1995). All handling of fishes was carried out in accordance with the guidelines on the care and use of animals for scientific purposes set up by the Institutional Animal Care and Use Committee (IACUC) of the Shanghai Ocean University (SHOU), Shanghai, China. This research was approved by the IACUC of SHOU.

### RNA extraction and real time qRT-PCR

Embryos were collected at timed developmental stages. Total RNA was extracted from whole embryos using TRIzol Reagent according to the manufacturer’s protocol (Invitrogen). For quantification of miRNAs, miRNA-specific stem-loop RT primers were designed with the software primer 5.0. The isolated RNA was reverse transcribed into cDNA by miRNA-specific stem-loop RT primers and PrimeScript® RT reagent Kit (Takara). qRT-PCR was performed using the miRNA-specific stem-loop RT primers and SYBR Green Master Mix following the manufacturer’s protocol (Takara). For quantification of pri-miR-202 and protein coding transcripts, total RNA from the embryos were reverse transcribed using random primers supplied in the PrimeScript® RT kit following the same protocol as above (Takara). All samples were performed in triplicates, and expression level of target genes was calculated with the 2^-△△CT^. U6 was used as the internal control. The primers used are listed in Supplementary Table S5.

### Microinjection of antagomirs of miR-202

Antagomirs to miR-202-3p, miR-202-5p and the scrambled antagomir (as negative control) were designed and synthesized by GenePharma (China). The sequences are provided in Supplementary Table S5. Fertilized eggs from wild type zebrafish at the one-cell stage were injected with 1 nl of each antagomir (8 µM) by using a microinjector (Eppendorf). The injected embryos were maintained at 28.5°C for development.

### *In vivo* miR-202 deletion by CRISPR-Cas9 system

Deletion of the miR-202 locus from the zebrafish genome was carried out using the CRISPR-Cas9 system. CRISPR-Cas9 target sites were designed using an online tool ZiFiT Targeter software (http://zifit.partners.org/ZiFiT). Two gRNAs were chosen to delete the miR-202 locus; the primers are listed in Supplementary Table S5. Capped Cas9 mRNA was synthesized *in vitro* by mMESSAGE mMACHINE T7 ULTRA kit (Ambion), and purified using RNeasy Mini Kit (Qiagen). gRNAs were synthesized using MAXIscript T7 kit (Ambion) following the manufacturer’s protocol and purified. Approximately 400 pg mRNA encoding Cas9 and 100 pg gRNA were injected into each embryo. The embryos were raised and maintained at 28.5°C.

### Screening for F0 miR-202 mutant zebrafish

To screen F0 miR-202 mutant zebrafish, genomic DNA was isolated from embryos produced by crossing microinjected F0 zebrafish with wild type partners. The target region was amplified by PCR using the specific primer pairs that were designed to distinguish wild type and mutated alleles (Supplementary Figure S1, Supplementary Table S5). The F0 parents who produced the miR-202 mutant embryos were identified. Mutation status of their miR-202 locus was further verified through PCR amplification and sequencing.

### Genotype identification of F2 embryos

The embryonic fatality of the homozygous miR-202 mutant rendered unavailable sexually mature miR-202^−/−^ individuals for reproduction. Therefore, investigation of miR-202^−/−^ phenotypes and underlying mechanisms relied on precise genotyping of embryos produced from heterozygous miR-202 parents. Genomic DNA was isolated from a single embryo using the alkaline lysis method: a timed embryo produced by heterozygous parents was submersed in 20 µl of 50 mM NaOH and heated to 95 °C for 10min. The tube was then vortexed and heated again, and 2 µl of Tris-HCl (1 M, pH = 8.0) was added to neutralize the solution. The tube was centrifuged and the supernatant was collect for PCR amplification with the proper primer (Supplementary Figure S1A). In the cases when embryos were taken prior to 10 hpf, the nested PCR was used for genotyping (Supplementary Figure S1B). The primers are listed in Supplementary Table S5.

### miR-202^−/−^ embryo rescue using synthetic agomirs

Agomirs for miR-202-3p, miR-202-5p and pre-miR-202 were chemically synthesized (Sangon Biotech) based on their native sequences (Supplementary Table S5). Series of dilutions of each agomirs or mixture or precusor (10 μM, 20 and 30 µM) were microinjected into the one-cell fertilized eggs obtained from the miR-202 heterozygous parents with 1 nL using a microinjector (Eppendorf). Developmental status of the injected embryos were observed real-time under a stereomicroscope (Zeiss). Embryos that survived to 12 hpf were picked out, counted, and genotyped.

### RNA-seq and analysis

Genomic DNA and total RNA were concurrently isolated from single embryo collected from mating of miR-202^+/-^ parents at 3.5 hpf. After genotyping of the embryos with DNA, the total RNAs from same-genotype embryos, namely miR-202 homozygous, or heterozygous, or wild type embryos were pooled to gain a sufficient amount of total RNA for each genotype for sequencing. RNA-seq was performed by NovoGenes (Tianjin, China). RNA-seq reads were trimmed using Trimmomatic (Bolger et al., 2014) (Ver. 0.33 AVGQUAL:20 TRAILING:20 MINLEN:50). The clean Illumina paired-end reads of each sample were mapped to the annotated zebrafish genome (GRCz10) using HISAT2 aligner (Kim et al., 2015) (Ver. 2.0.4). Cufflinks was used to count the reads for each gene and transformed to FPKM. Differentially expressed genes (DEGs) between the genotypes were determined using the edgeR (Robinson et al., 2010) package developed in R. Compare homo_3.5 h with hete_3.5 h and wt_3.5 h, respectively, for log2 fold change >1 or < −1 and *p*_value <0.05 was defined as differentially expressed genes (DEGs). DEGs related with the maternally inherited mRNAs were identified by adopting the following criteria: 1) if FPKM (wt_0 h≥homo_3.5 h≥max (hete_3.5h, wt_3.5 h), the gene was taken to indicate insufficient degradation (ID); 2) if FPKM (homo_3.5 h > max (hete_3.5h, wt_3.5h, wt_0 h), the gene was associated with over-expression (OE); and 3) if FPKM (min (hete_3.5h, wt_3.5 h)≥homo_3.5 h≥wt_0 h), the gene was regarded as insufficient expression (IE). Almost no genes were over degraded in miR-202^−/−^ embryos and were thus not considered for GO and KEGG enrichment.

### Proteomic analyses of the miR-202 mutant and wild type embryos

The wild type and abnormally developing embryos of miR-202^+/-^ pairs were collected at 4hpf, and the embryos were removed from the egg shell. Three groups of normal WT (A_4hpf) and three groups of abnormal embryo (D_4hpf) samples (each having about 50 embryos) were used to extract protein for proteomic analysis. Proteomic analysis was performed using LC-MS/MS on a QExactive mass spectrometer with an Easy-nLC system (Thermo Fisher Scientifc). The LC-MS/MS data were analyzed using Proteome Discovery (Version 2.2, Thermo Fisher Scientific) with the zebrafish Uniprot database (uniprot-danio + rerio_170221.fasta). To quantify protein, the abundance value was normalized with the median value of the whole protein set and only unique peptides were used. Differential protein screening was performed at criteria of 1.2 and 0.833 fold change (FC).

### Whole mount *in situ* hybridization

A digoxigenin (DIG) labelled RNA probe of miR-202-3p (accession number: MIMAT0001864) and a scrambled RNA probe (NC) were synthesized by Exiqon (Denmark). Whole mount *in situ* hybridization (WISH) was performed as previously described (Thisse et al., 2008). Shell-removed embryos were fixed in 4% PFA (paraformaldehyde) at 4°C overnight, dehydrated in methanol and rehydrated by a series of methanol/PBST gradients, and then treated with proteinase K and re-fixed in 4% PFA. Embryos were pre-hybridized with hybridization mixture (HM) at 58°C for 2–4 h and hybridized with DIG-labelled miR-202-3p anti-sense probe or scrambled probe (NC) at 58°C overnight. After hybridization, embryos were washed in a series of saline sodium citrate (SSC) gradients. Subsequently, the embryos were blocked in MAB buffer with 1% blocking solution (Roche) for 3 h at room temperature and incubated in alkaline phosphatase conjugated anti-DIG antibody (1:5,000 diluted in blocking solution, Roche) at 4°C overnight. The embryos were washed four times in PBST for 15 min, and the signal was developed using NBT/BCIP Staining solution. The images were documented with a stereomicroscope (Zeiss) equipped with a digital camera.

### *In situ* hybridization of tissue sections

*In situ* hybridization on paraffin sections was performed as described previously (Jørgensen et al., 2010). Briefly, ovary tissue or embryos were dissected in 1 × PBS and fixed in 4% PFA overnight at 4°C. Fixed tissues were embedded in paraffin and sectioned using a paraffin slicer microtome (Leica) at 10-µm thickness and transferred to special coating glass slides (Leica). Slides were hybridized overnight with 1 μg/ml digoxigenin-labeled probe at 65°C in HM solution. After washing in SSC buffer, slides were incubated with alkaline phosphatase-coupled anti-digoxigenin antibodies overnight at room temperature. Slides were then dehydrated through ethanol series and xylene (Sigma-Aldrich) then mounted using Entellan (Electron Microscopy Sciences). Images were acquired using a confocal microscope (Zeiss).

### Protein translation efficiency assay

F1 heterozygous zebrafish adults were crossed in the appropriate breeding tanks. EGFP mRNA was transcribed *in vitro* as above. The fertilized eggs (one-cell stage) were collected immediately and injected with EGFP mRNA (100 pg) and then cultured at 28.5°C for 3 hours. Embryos were screened by fluorescence analysis with a stereomicroscope (Zeiss). Embryos with bright and weak green fluorescence intensities were selected for genotyping verification.

### Cellular ROS detection

F1 heterozygous zebrafish adults were crossed in the appropriate breeding tanks. Fertilized eggs were collected and stored at 28.5°C to allow the embryos to develop for 3 hours. Embryos were then washed with E3 medium (Cold spring Harbor Protocols 2011, pdb. rec66449, doi: 10.1101/pdb.rec066449 (2011)) and then immediately incubated with a general Oxidative Stress Indicator (CM-H2DCFDA) (Invitrogen) at a final concentration of 3 µM. Embryos were incubated in the dark for 15 min at 28.5°C. At the end of the incubation, the ROS-detection solution was immediately removed and embryos were washed three times with E3. Fluorescence intensity of the embryos was analyzed by stereomicroscope (Zeiss). Embryos with bright and weak green fluorescence were selected for genotyping verification.

### SYTOX staining

SYTOX nuclear green stain is impermeable to living cells, but stains nuclei in a syncitium (or otherwise following membrane degradation) (Goonesinghe et al., 2012). To visualise migration of YSL nuclei relative to the blastoderm margin during epiboly, embryos from heterozygous parents were injected with 1 nl of 0.5 mM Sytox Green fluorescent nucleic acid dye (Invitrogen, United States) into the yolk cell at 3 hpf and then visualised at 4 hpf and 6 hpf under a fluorescence stereomicroscope (Zeiss). Embryos were kept in E3 medium for genotyping verification. Images were captured and processed using a Zeiss AxioCam MR and AxioVision 4.5 software.

### Cell adhesion detection

F2 embryos from heterozygous parents were used for immunofluorescence staining. Embryos were fixed overnight in 4% PFA at 4°C, and then were peeled off the egg shells. Embryos were permeabilized in 0.5% Triton-X-100 for 30 min at room temperature. After 1 h blocking in 1% BSA/PBS at room temperature, embryos were incubated overnight at 4 °C with primary antibody anti-ZO-1 at 1:200 (Thermal Fisher Scientific). After three washes, embryos were incubated with secondary antibody at 1:3,000 (Thermal Fisher Scientific) for 2 h at room temperature. TSA-F green fluorescent dye staining (1:100) was used to amplify signals by incubation at room temperature for 30 min in the dark, and then washed with PBS for at least 1 h. DAPI (500 ng/ml) was added to counterstain the nuclei followed by washing with PBS for three times. Photographs were taken using confocal microscopy (Zeiss). Following photography, the embryos were then genotyped individually.

### Apoptosis detection

F2 embryos from heterozygous miR-202 parents were used for TUNEL staining. Embryos were fixed in 4% PFA at 4°C overnight, then removed egg shell from embryos. TUNEL staining was performed using a commercially available kit (Thermo Fisher Scientific) by following the manufacturer’s instructions. The embryos were stained with FITC-dUTP Labeling Mix and DAPI (500 ng/ml), then were analyzed under a laser confocal microscope (Zeiss). After being photographed, each embryo was genotyped.

### Phenotype rescue using shRNAs of miR-202-3p target genes

To validate the function of the miR-202-3p target genes in the embryonic lethal phenotype of miR-202-3p deficiency, shRNAs against the Seven genes (*nfkbiaa*, *perp*, *mgll*, *atp1b1a*,*nfil3-5*, *pleca*, *nfe2l2b*) which were predicted to be miR-202-3p targets and upregulated in the miR-202^−/−^ embryos were designed through the BLOCK-iT™ RNAi Designer (Thermo Fisher Scientific), chemically synthesized (Sangon Biotech) and cloned to pLKO.1 plasmid (Addgene). shRNA plasmid was microinjected into WT embryos together with the miR-202-3p antagomir, in a final concentration of 200 ng/μL and 8 nM, respectively. Then injected embryos were raised and maintained at 28.5 °C and observed for developmental status; rescue rate was calculated for every 2 h. The shRNAs are listed in Supplementary Table S5.

### Knockdown and overexpression of miR-202-3p target genes in developing embryos

We manipulated the mRNA contents of miR-202-3p target genes, *nfkbiaa*, *perp* and *mgll* in developing embryos for validating the function of these genes in embryonic development. To down-regulate a gene, a single type or a mix of the plasmid constructs containing specific shRNA was microinjected into the wild type embryos, in a final concentration of 200 ng/μl, and 100 ng/μl for each one in the mixture. To increase the mRNA content of a specific gene in developing embryos, mRNA was microinjected into wild type embryos with final concentration of 400 ng/μl for a single gene or 200 ng/μl for each one in the mixture. Each embryo was injected in 1 nl volume. Embryos after injection were cultured at 28.5°C and observed for developmental status every hour. The shRNA sequences and primers for cDNA amplification are listed in Supplementary Table S5.

### Embryo viability statistics

The developmental stages of embryos were examined using a stereomicroscope (Zeiss) by observing the morphological appearance. An embryo was considered to be dead if lysed cells were visible under the microscope. An embryo was considered to be abnormal if development was slower than in the wild type, and developmental termination was registered for an embryo if no morphological progression was observed within a period of 1 hour. Rescue rate is calculated through a two step procedure: 1) counting all live embryos at 12 hpf and genotyping each embryo; 2) calculating the ratio of the living miR-202^−/−^ embryos to the total number of embryos examined. The ratio is regarded as the rescue rate of a reagent because through large scale phenotype and genotype analyses, we had established that no miR-202^−/−^ embryos would survive beyond 12 hpf without rescue.

### Target gene validation through dual luciferase assay

Dual luciferase assay was carried out to validate the authenticity of the predicted target genes of miR-202-3p. Native and mutated 3′UTRs of the candidate genes were amplified from zebrafish embryonic cDNA and cloned into the pmirGLO Dual-Luciferase miRNA Target Expression Vector (Promega) and sequenced. HEK293T cells were plated in a 96-well plate and incubated at 37°C for 24 h miR-202-3p agomir (or scrambled agomir) and pmirGLO-3′UTR (or mutated 3′ UTR) construct were co-transfected into the HEK293T by using Attractence Transfection Reagent (QIAGEN). The transfected Cells were continuously incubated at 37 °C for 24 h, and luciferase activity was measured using the Dual-Luciferase Reporter Assay System (Promega) following the manufacturer’s protocol in a luminometer. Data were first normalized to residual luminescence then to an agomir negative control. The 3′UTRs or mutation sites and primers are shown in Supplementary Figure S3 and Supplementary Table S5.

## Imaging

Embryos were treated with 0.02% tricaine (3-amino benzoic acid ethyl ester), mounted in 3% methyl-cellulose, and visualized under a Stereoscopic Microscope (Zeiss).

### Statistical analysis

Statistical analysis was conducted using the Student’s t-test (two-tailed). All values are shown as mean ± s.d. *p* < 0.05 were considered statistically significant. One asterisk, two asterisks and three asterisks indicate < 0.05, *p* < 0.01 and *p* < 0.001, respectively.

## Results

### Inhibition of miR-202-3p but not miR-202-5p terminates embryonic development at mid-blastula stage

**FIGURE 1:**
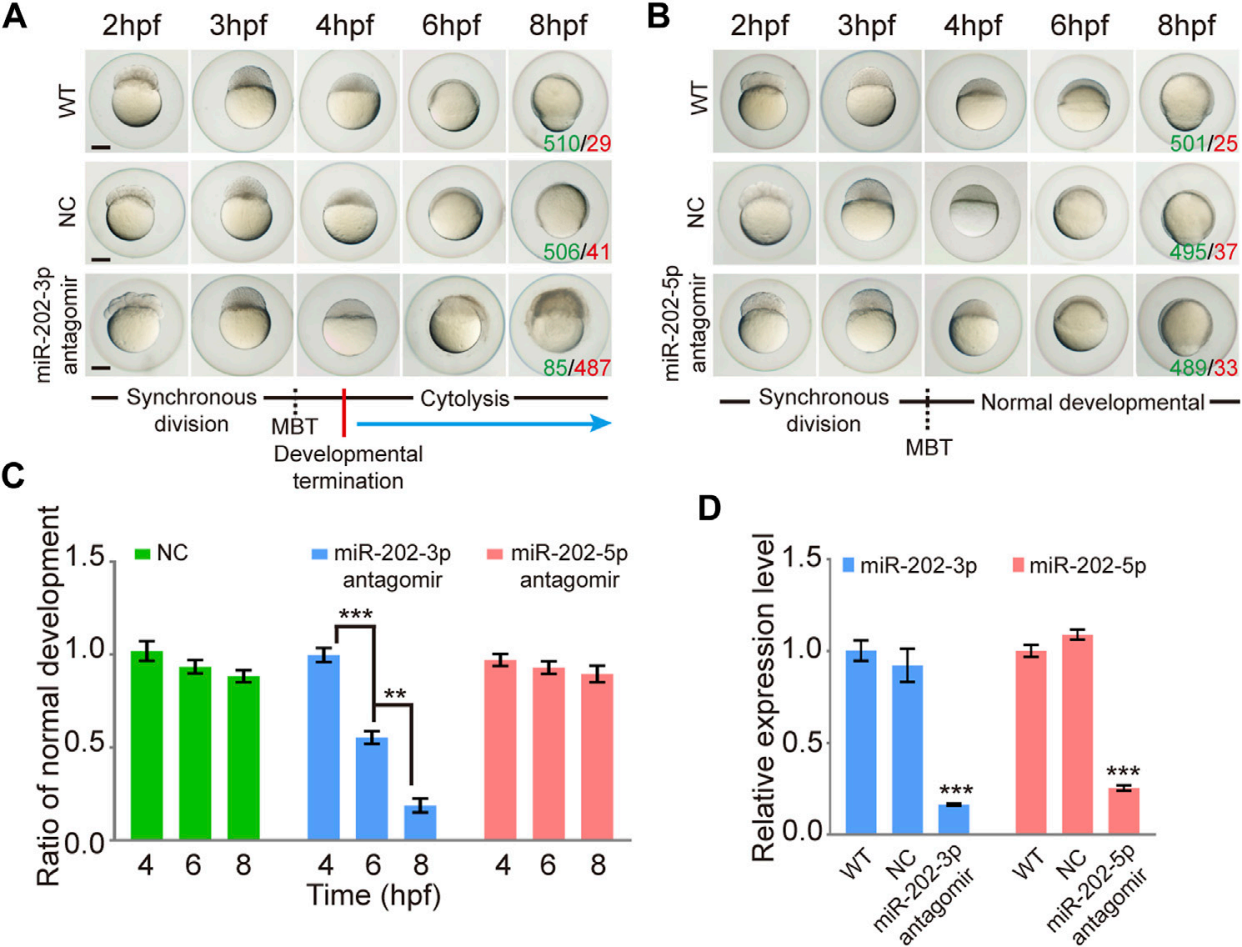
Inhibition of miR-202-3p by an antagomir results in termination of embryonic development at MBT in zebrafish. **(A)** Time-matched bright field images of embryos showing developmental termination at 4 hpf and, finally, cytolysis after miR-202-3p antagomir (8 µM) injection in the fertilized embryos compared with embryos of wild type and NC embryos injected with scrambled miR-202-3p antagomir (8 µM). The green number indicates the number of embryos with normal development and in red the number indicates the abnormal development. WT embryos at 8hpf were used as phenotypic control. **(B)** Time-matched bright field images of embryos showing completely normal development of zebrafish embryos injected with miR-202-5p antagomir (8 µM); patterns are similar with what is found for wild type (WT) and NC (scrambled antagomir) injection embryos over the same developmental time course. The green number indicates the number of embryos with normal development and in red the number indicates the abnormal development. WT embryos at 8hpf were used as phenotypic control. **(C)** The statistics of embryo viability at 4 hpf, 6 hpf and 8 hpf following injection with miR-202-3p antagomir, miR-202-5p antagomir or NC (scrambled antagomir). **(D)** qRT-PCR analyses showing embryonic content of miR-202-3p and miR-202-5p after treatment with respective antagomirs. The scale bar is 100 µm. Error bars, mean ± s.d., n = 3 (biological replicates).

To test whether miR-202-3p and miR-202-5p play a role in early embryonic development, we microinjected the antagomirs of miR-202-3p and miR-202-5p respectively into fertilized embryos. Injected embryos developed normally at the initial stages, similar with the control group (injected with a scrambled antagomir) and the wild type embryos. However, starting from 4 hpf, embryos injected with the miR-202-3p antagomir demonstrated developmental stoppage and the blastomere disassociated from 6 hpf to 12hpf (Figure 1A). Once the threshold amount of miR-202-3p antagomir (8 µM) was reached, developmental failure occurred in over 90% of the embryos during the blastula stage. In sharp contrast, no developmental abnormality was observed in the embryos injected with the miR-202-5p antagomir or the control antagomir at the same amounts (Figure 1B). The time course statistics for blastomere cytolysis in injected embryos are shown in Figure 1C, which is based on more than two thousand injected embryos for each antagomir. qRT-PCR was performed to evaluate the efficiency of miRNA knockdown with their respective antagomirs; both miR-202-3p and miR-202-5p antagomirs functioned effectively (Figure 1D). These results suggested that miR-202-3p might be essential for embryonic development at the mid-blastula stage.

### Deletion of miR-202 in zebrafish using CRISPR-Cas9

**FIGURE 2:**
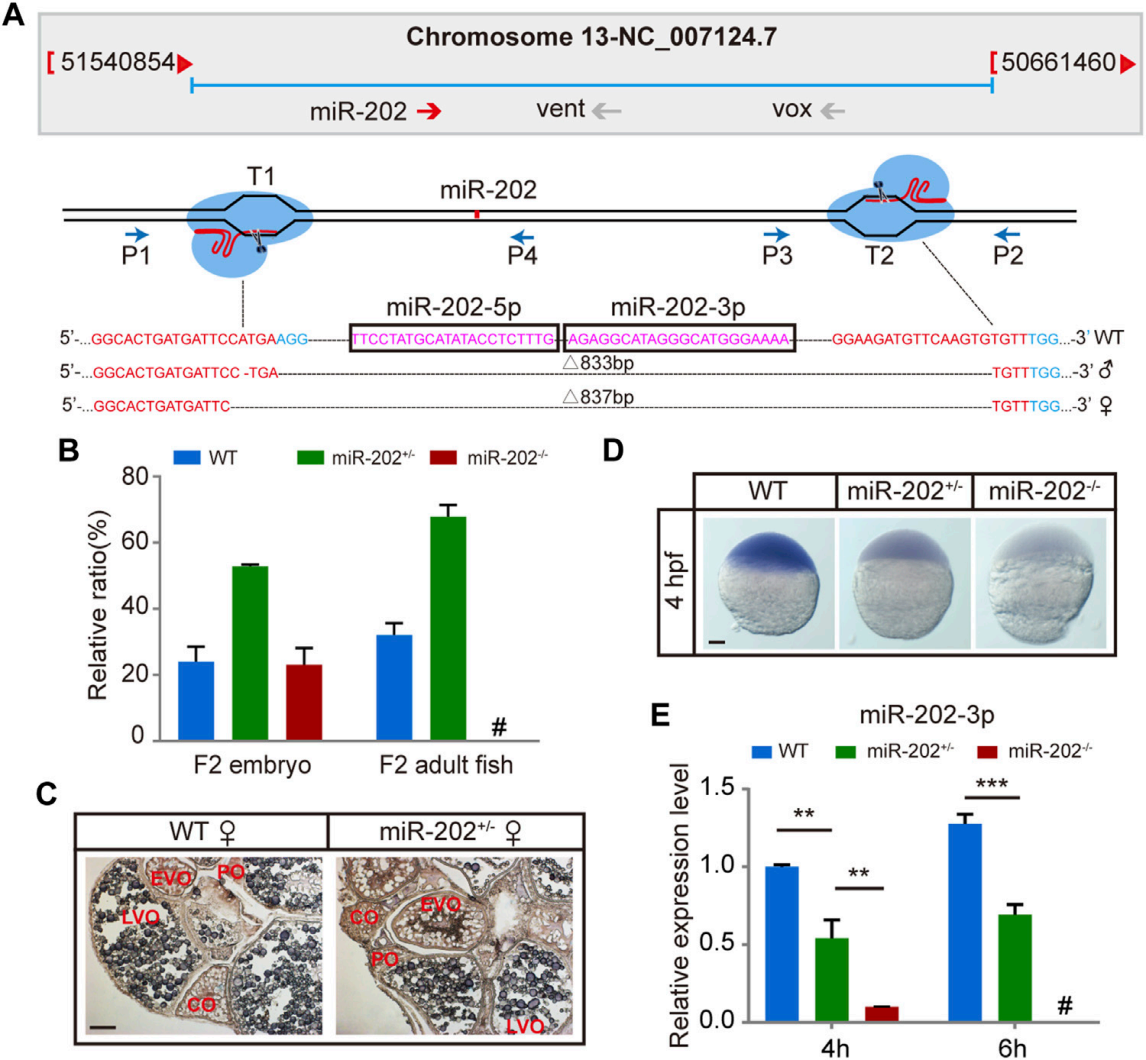
Deletion of miR-202 from the zebrafish genome using CRISPR-Cas9 system. **(A)** Schematic illustration of CRISPR/Cas9 system used to produce the knock-out lines of miR-202. The miR-202 sequences are in magenta, miR-202-3p and miR-202-5p are marked with a rectangular frame; the sgRNA target sites are in red; the PAM motif (NGG) is shown in blue. The location and direction of primers (P1-P4) used for PCR screening are also shown with arrow. **(B)** Genotyping of miR-202 mutant F2 embryos at 4hpf and F2 adults (3 month). **(C)** *In situ* hybridization of ovaries from miR-202 wild type (WT) and heterozygotes (miR-202^+/-^) to examine expression of miR-202-3p. The developmental stages of ovarian oocytes include the primary oocytes: perinuclear oocytes (PO) and cortical alveolar oocytes (CO); the mature oocytes: early vitellogenin oocytes (EVO) and late vitellogenin oocytes (LVO). The scale bar is 10 µm. **(D)** Whole mount *in situ* hybridization showing expression of miR-202-3p in wild type (WT), heterozygous (miR-202^+/-^) and homozygous (miR-202^−/−^) embryos at 4 hpf. The scale bar is 100 µm. **(E)** qRT-PCR analysis of miR-202-3p expression in wild type, heterozygous and homozygous embryos at 4 hpf and 6 hpf. Error bars, mean ± s.d., n = 3 (biological replicates).

To ensure that the blastula lethality phenotype that occurred in the miR-202-3p antagomir injected embryos is an authentic function of this miRNA, we targeted the miR-202 locus for deletion using the CRISPR-Cas9 technology in zebrafish. Two founders (a male and a female) of zebrafish with deletions of 833 bp or 837 bp fragment were generated (Figure 2A). Heterozygous F1 lines were generated by mating the two founders to wild type female or male fish. The miR-202^+/-^ fishes developed normally like the wild type fish. However, the genotype of F2 embryos were inherited at the expected Mendelian ratio at 4 hpf, while no zygous miR-202 deletion zebrafish can survive to adulthood, indicating that miR-202 is crucial for zebrafish development (Figure 2B). *In situ* hybridization of ovary sections prepared from the wild type and miR-202^+/-^ fishes showed that in both fishes, miR-202-3p was expressed in the developed oocytes with similar intensities but absent from the underdeveloped oocytes (Figure 2C), further suggesting maternal carryover of miR-202-3p at the initial developmental stages. By cross-mating between the miR-202^+/-^ F1 fishes, developmentally timed F2 embryos were collected at 4 hpf. Whole mount *in situ* hybridization on these embryos demonstrated differential staining intensities for miR-202-3p, corresponding to the wild type, miR-202^+/-^ and miR-202^−/−^ genotypes, respectively (Figure 2D). qRT-PCR analyses on genotyped embryos clearly indicated reduced miR-202-3p expression in the miR-202^+/-^ embryos and only residual levels of miR-202-3p in the miR-202^−/−^ embryos at 4 hpf, which was carried over from the oocyte. However, it could not detect the miR-202-3p in the miR-202^−/−^ embryos at 6 hpf (Figure 2E).

### Deletion of the miR-202 locus recapitulated the fatal phenotype of miR-202-3p inhibition

**FIGURE 3:**
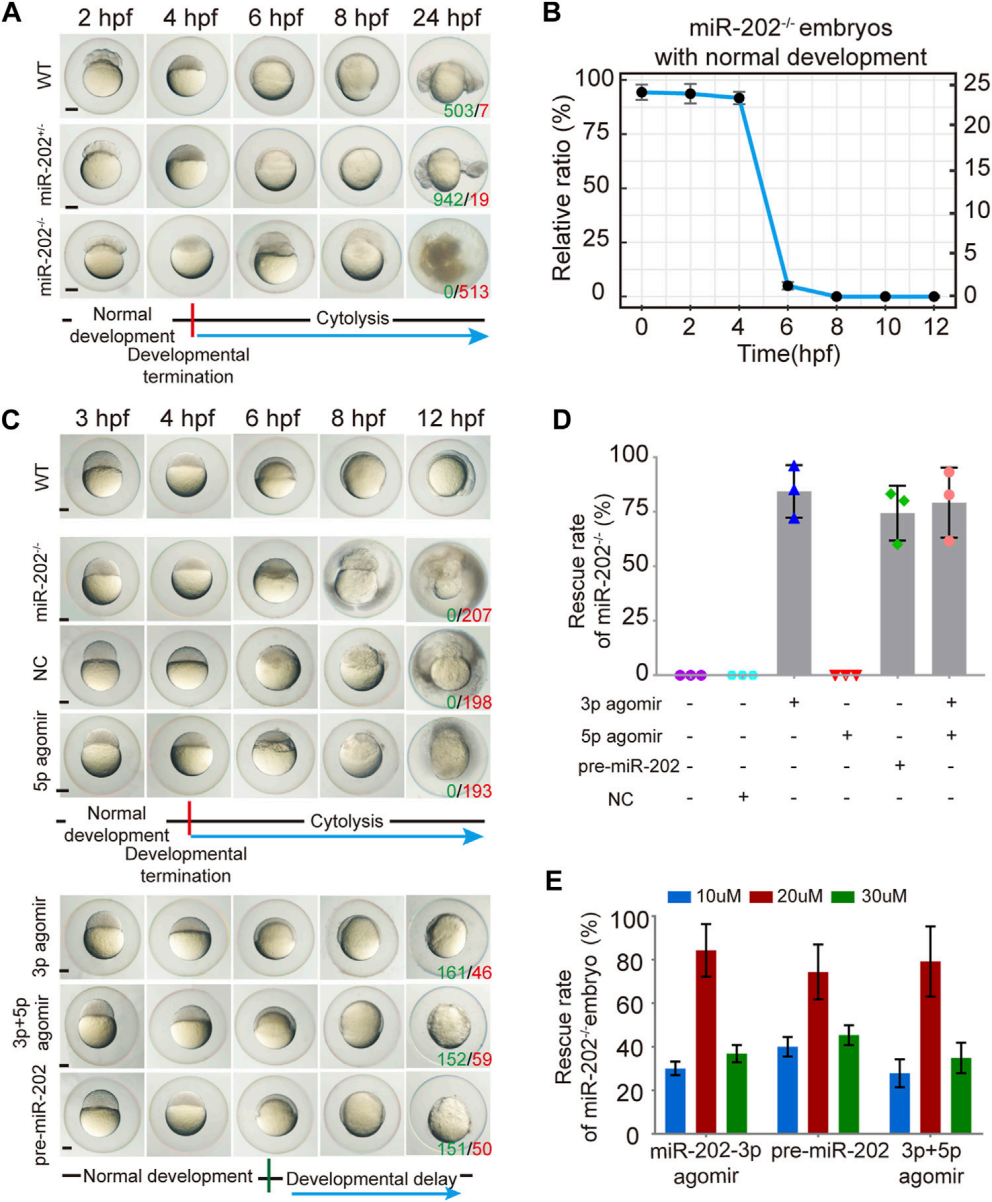
Deletion of the miR-202 locus recapitulated the phenotype of miR-202-3p knockdown. **(A)** Time-matched bright field images showing developmental termination of homozygous (miR-202^−/−^) embryos at 4 hpf in contrast with normal development of heterozygous (miR-202^+/-^) and wild type (WT) embryos. The green number indicates the number of embryo with normal development and in red the number indicates the abnormal development. WT embryos at 24 hpf were used as phenotypic control. **(B)** The survival rate curve for miR-202^−/−^ embryos from 0 to 12 hpf. **(C)** Time-matched bright field images of miR-202^−/−^ embryos when rescued by various reagents. The green number indicates the number of embryo that has been rescued and in red the number indicates the unsalvaged. 3p agomir embryos at 12 hpf were used as the control of successful rescue phenotype. **(D)** Rescue rates of miR-202^−/−^ homozygotes by miR-202-3p agomir, miR-202-5p agomir, pre-miR-202 and a combination of miR-202-3p and miR-202-5p agomirs measured at 12 hpf. **(E)** The rescue rates of miR-202^−/−^ embryos obtained with three different concentrations (10 μM, 20 and 30 µM) of rescue reagents measured at 12 hpf, indicating an optimal rescuing concentration for each reagent. The scale bar is 200 µm. Error bars, mean ± s.d., n = 3 (biological replicates).

The miR-202^+/-^ F1 females were paired with F1 miR-202^+/-^ males to examine the viability of embryos. While the majority of F2 embryos developed normally, about 1/4 stopped development and become visibly abnormal starting from 4 hpf, followed by cytolysis within next few hours (Figure 3A). This fatal phenotype highly resembled that of the embryos in which miR-202-3p is knocked down in the timing of occurrence and severity of the phenotype. In the more than 2000 F2 embryos genotyped, more than 90% of miR-202^−/−^ embryos demonstrated developmental termination and cytolysis within 4–6 hpf, and no miR-202^−/−^ embryos survived beyond 12 hpf (Figure 3B). No developmental abnormality occurred in the miR-202^+/−^ embryos at this stage.

To verify whether the fatal miR-202^−/−^ phenotype was a bona fide outcome from miR-202 deletion, we performed rescue attempts by injecting agomir of miR-202-3p, miR-202-5p, pre-miR-202, a combination of miR-202-3p and miR-202-5p, and a scrambled miR-202-3p (i.e., NC) to the F2 embryos. By extensive genotyping of viable embryos at 4, 6, 8, 10, and 12 hpf for each rescue agent, we found 80 ± 12%, 75 ± 12% and 77 ± 16% of miR-202^−/−^ embryos demonstrated delayed development not the cytolysis at the 12 hpf time point for miR-202-3p, pre-miR-202, the combination of miR-202-3p and miR-202-5p, respectively, while none of the miR-202^−/−^ embryos were rescued by miR-202-5p and the scrambled miRNA (Figures 3C,D). Thus the fatal phenotype of miR-202^−/−^ at the blastula stage could be rescued if miR-202-3p is present in the rescue agents. Rescuing efficiencies of miR-202-3p and pre-miR-202 both peaked at 20 μM, with under- and over-doses resulting in two to three folds lower rescue rates (Figure 3E). Interestingly, both overdosed and inadequate amounts of miR-202-3p resulted in severely delayed embryo development and embryos underwent cytolysis at the blastula stage. These results indicate that miR-202-3p is required for embryonic viability and its expression be precisely regulated during MBT.

### Loss of miR-202 altered embryonic mRNA repertoire during MBT

**FIGURE 4:**
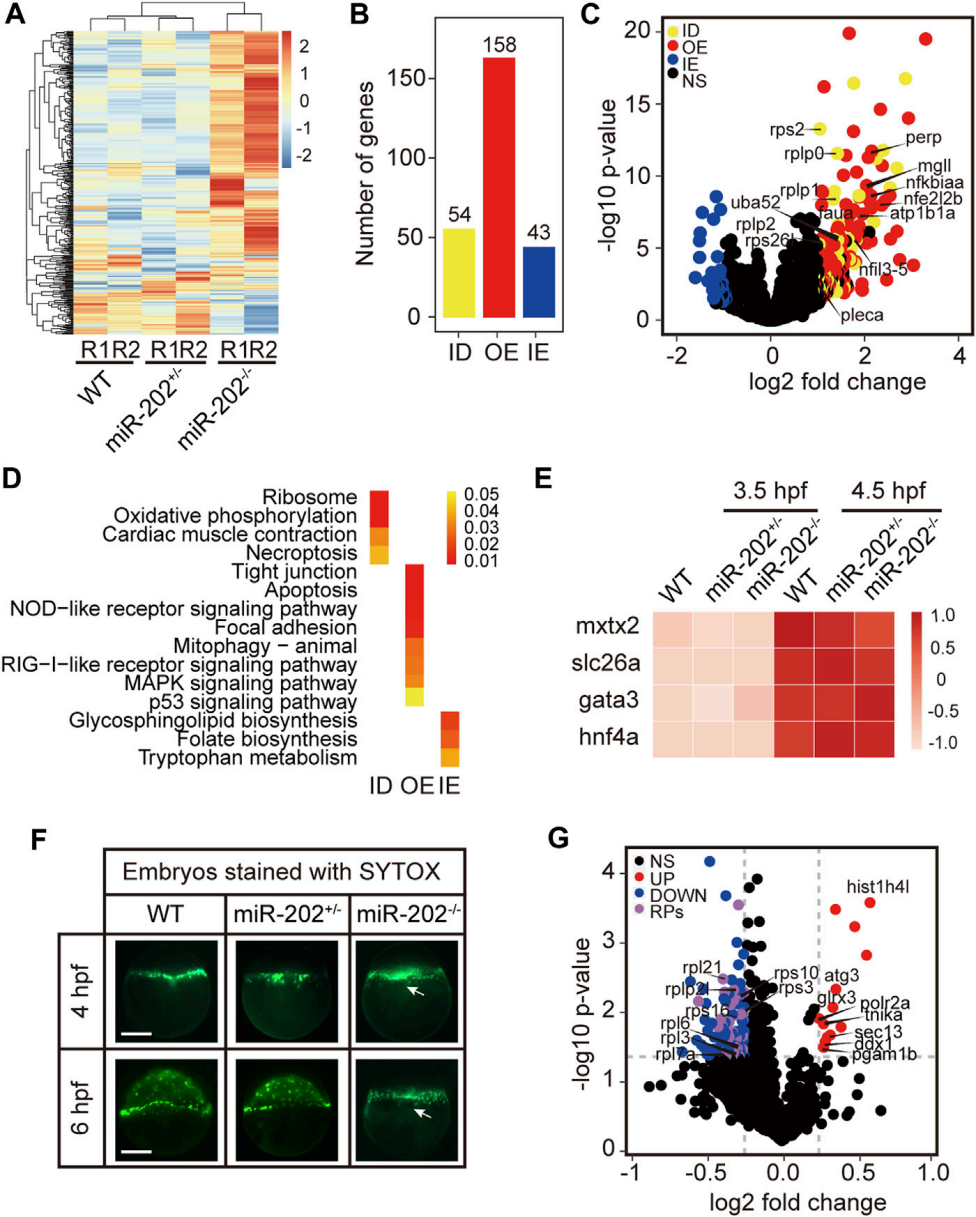
Transcriptomic and proteomic analysis for miR-202 mutant embryos. **(A)** Heat map showing distinctive gene expression patterns of miR-202^−/−^, miR-202^+/-^ and WT embryos at 3.5 hpf. R1 and R2 indicate sampling replicates. **(B)** The number of differentially expressed genes in three subgroups: ID, insufficient degradation; OE, over-expression; IE, insufficient expression. **(C)** Volcano plot of differentially expressed genes between homozygous and heterozygous miR-202 embryos. **(D)** KEGGs enriched in the three subgroups of differentially expressed genes. **(E)** Expression of genes related to the initiation and subcapsulation of Yolk Syncytial Layer (YSL) at 3.5 and 4.5 hpf in wild type and miR-202^+/-^ and miR-202^−/−^ indicating normal expression. **(F)** Verification that the loss of the miR-202 locus cannot affect the formation of YSL at 4 hpf, but miR-202^−/−^ embryos cannot successfully achieve epibolic movement compared with wild type and miR-202^+/-^ embryos. The white arrow represents YSL. The scale bar is 200 µm. **(G)** Distribution of differentially expressed proteins in miR-202^−/−^ embryos. NS, no significant difference; UP, up-regulated; DOWN, down-regulated; RPs, ribosome-associated proteins.

To elucidate mechanisms of miR-202-3p function in early development, we conducted transcriptome comparisons among the WT, miR-202^+/−^, and miR-202^−/−^ embryos collected at 3.5 hpf. RNA and DNA from single embryos were isolated concurrently and genotyping was carried out using the DNA. RNA from embryos of same genotype was pooled for RNA-seq. As expected, gene expression profiles from the miR-202^−/−^ embryos showed greater divergence from the heterozygous and wild type embryos while the latter two are more similar (Figure 4A). The expression levels of the DEGs were then compared with wild type embryos at the one-cell/fertilized egg stage to determine how the DEGs were related with the maternally inherited mRNAs. This comparison allowed us to divide the DEGs into three subgroups: insufficient degradation (ID) of maternal RNA, overexpression (OE) of zygotic genes, and insufficient expression (IE) of zygotic genes in the miR-202^−/−^ embryos (Figure 4B, Supplementary Table S1). There are 54 genes including 7 ribosomal proteins that were insufficiently degraded, 158 genes, such as *nfkbiaa*, *perp*, *mgll* involved in cell proliferation, apoptosis and cell-cell adhesion are over-expressed, and 43 genes insufficiently expressed (Figure 4C). We further analyzed the three subgroups of DEGs for KEGG enrichment; 15 pathways including ribosome, oxidative phosphorylation, apoptosis, cell junction, inflammatory and metabolic related pathways were identified (Figure 4D, Supplementary Table S2). A larger portion of DEGs (63%) belong to the over-expressed subgroup, suggesting a suppressive role of miR-202 during zygotic genome activation. Due to failure in the initiation of epibolic movement in miR-202^−/−^ embryos, we are curious whether loss of miR-202 would affect the formation the Yolk Syncytial Layer (YSL), the structure critical for initiating epibolic cell movement and reorganization. We found transcription of the YSL marker genes (Xu et al., 2012) was normal in miR-202^−/−^ embryos (Figure 4E). Staining of the F2 embryos using SYTOX also showed proper formation of YSL in all embryos including the miR-202 mutants at 4 hpf. However, with development progressed, YSL in the wild type embryos was shown to have a complete epibolic movement of cells towards the vegetal pole, but epibolic movement failed in the miR-202^−/−^ embryos, which clearly pinpointed the timing of developmental termination was prior to epiboly in the mutant (Figure 4F).

To evaluate the proteomic consequences of miR-202 deletion, we analyzed the proteome of miR-202^−/−^ embryos and compared it to the wild type at 4 hpf. We observed a total of 115 differentially expressed proteins, of which 100 were down-regulated and 15 were up-regulated in miR-202^−/−^ embryos (Supplementary Table S3). A most peculiar feature of the miR-202 null embryonic proteome is the reduction of 34 ribosomal proteins (Figure 4G, Supplementary Table S3).

### Homeostatic disorders and embryonic apoptosis in miR-202 deletion embryo

**FIGURE 5:**
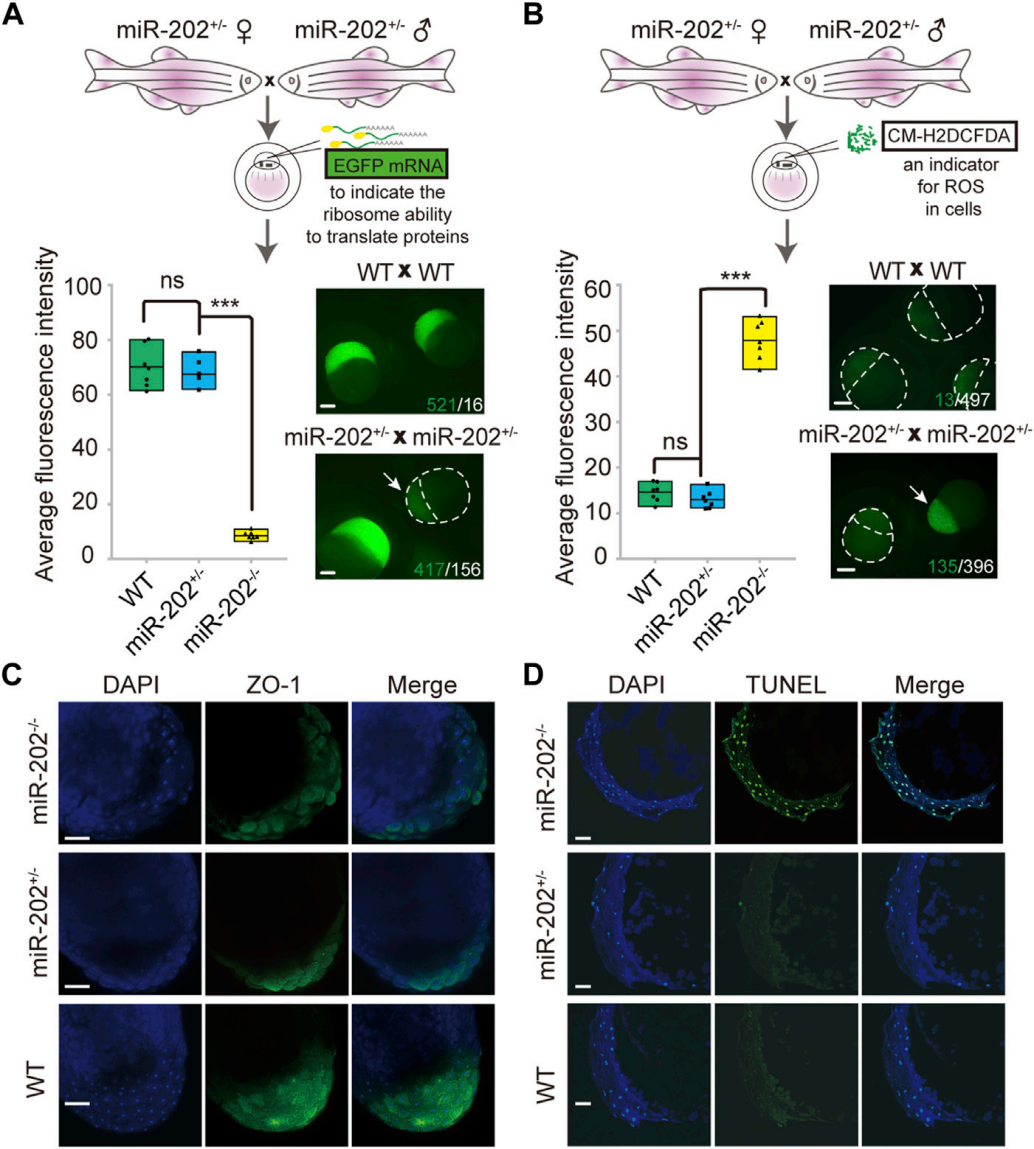
Homeostatic disorders in miR-202^−/−^ embryos. **(A)** Illustrations showing assessment of translation efficiency using polyadenylated EGFP mRNA. Embryos derived from miR-202 heterozygous parents were used for assessment. Drastically reduced fluorescence intensity (bottom left panel) and images (bottom right panel) were obtained from the miR-202^−/−^ embryos at 3.5 hpf. The embryo pointed to by white arrows was a miR-202^−/−^ embryo, as verified through genotyping. The scale bar is 200 µm. The green number indicates the number of embryos successfully expressing EGFP, and in white the number indicates the number of embryos that unexpressed or low expressed EGFP. **(B)** Illustrations showing assessment of reactive oxygen species using CM-H2DCFDA. Embryos derived from miR-202 heterozygous parents were used for assessment. Elevated fluorescence intensity (bottom left panel) and images (bottom right panel) were obtained in the miR-202^−/−^ embryos at 3.5 hpf. The embryo indicated by the white arrow was a miR-202^−/−^ embryo, as verified through genotyping. The green number indicates the number of embryos that produce high level of ROS, and in white the number indicates the number of embryos with low ROS levels. The scale bar is 200 µm. **(C)** Immunohistochemical detection of zonula occludens-1 (ZO-1) in wild type and miR-202 mutant embryos at 3.5 hpf. The scale bar is 100 µm. **(D)** TUNEL staining for apoptotic signals in the wild type and miR-202 mutant embryos at 3.5 hpf. The scale bar is 100 µm. Error bars, mean ± s.d., *n* = 3 (biological replicates).

The identification of multiple dysregulated KEGG pathways from the transcriptome comparisons prompted us to evaluate the cellular consequences that the loss of miR-202 might produce. Due to the striking presence of dysregulated ribosomal proteins in the transcripts and proteome, we first verified whether protein synthesis was impaired. Exogenous introduction of polyadenylated EGFP mRNAs to the embryos produced by miR-202^+/-^ parents showed almost complete abolishment of protein synthesis in the miR-202 null embryos (Figure 5A). The level of reactive oxygen species (ROS) was also measured to evaluate whether oxidative stress is an outcome of the dysregulated pathways. We microinjected CM-H2DCFDA, an indicator of cellular ROS level into the embryos from the miR-202^+/-^ parents. About 4-fold higher ROS intensities were detected in the miR-202 null embryos (Figure 5B). In addition, cell-cell adhesion was also affected as demonstrated by the significantly reduced presence of zonula occludens-1 (ZO-1), the marker for tight junction (Figure 5C).

Overexpression of the apoptotic pathway in the miR-202^−/−^ embryos hinted that apoptosis could be the ultimate fate of the miR-202^−/−^ embryos. We performed TUNEL staining on sections of the F2 embryos followed with genotyping of the embryos. Apoptotic signals were widely detected from the blastomere cells in the miR-202^−/−^ embryos as compared to no detected signals in the miR-202^+/-^ and wild type embryos (Figure 5D). More than 90% of the cells in the blastomere at 3.5 hpf were visibly undergoing apoptosis in the miR-202^−/−^ embryos. Taken together, results strongly indicated that miR-202, through its precisely controlled product miR-202-3p, is essential to maintain cellular homeostasis during MBT.

### Screening for the target genes of miR-202-3p

**FIGURE 6:**
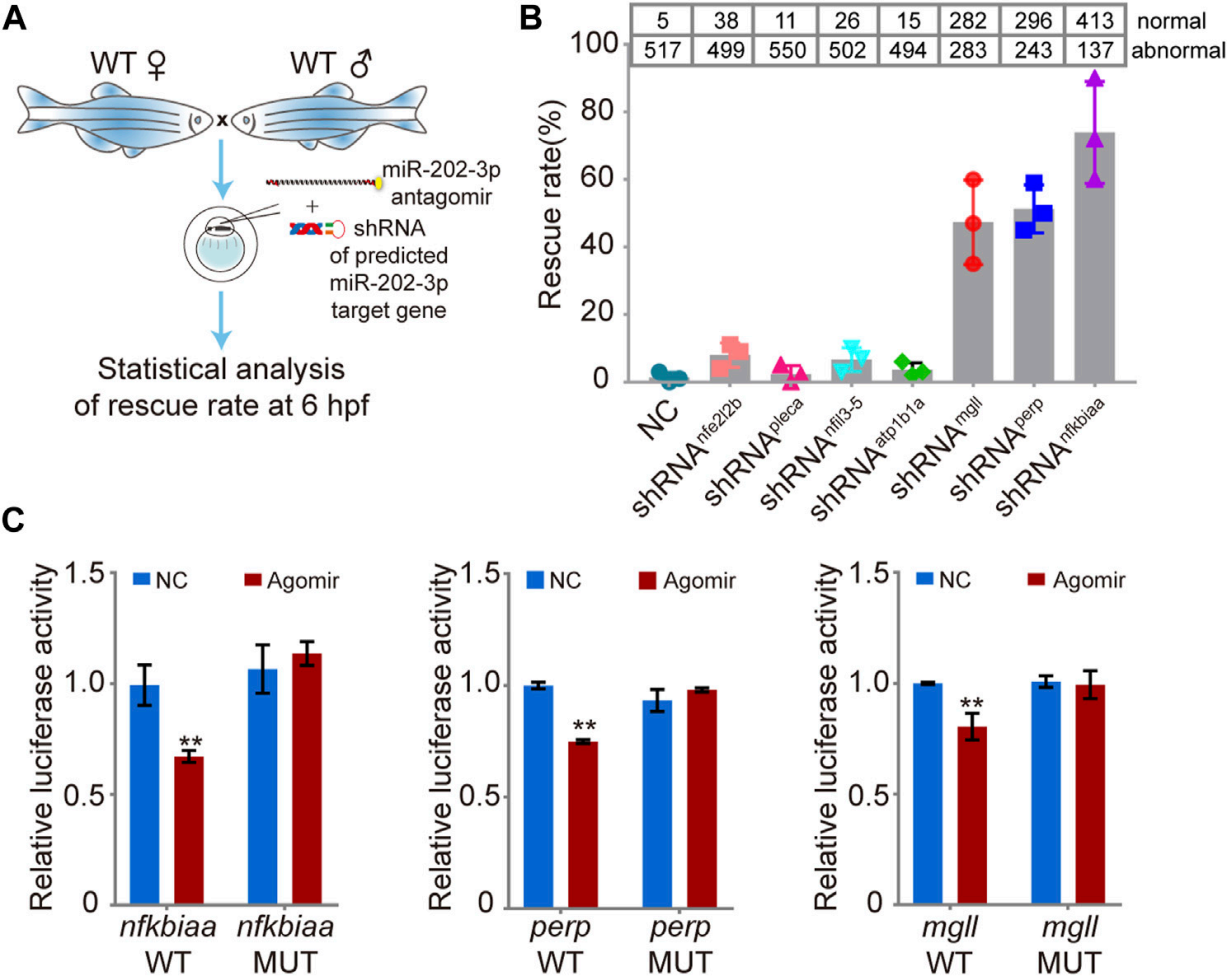
Validation of the function of the miR-202-3p target genes in embryogenesis. **(A)** Schematic illustration of rescue using shRNAs specific to the miR-202-3p target genes. Briefly, survival rates at 6 hpf were measured for wild type embryos co-injected with miR-202-3p antagomir (8 µM) and the specific shRNA (200 ng/ul) to one of the target genes at the 1-cell stage. **(B)** Rescue rates at 6 hpf for various shRNAs tested. More than 500 embryos were detected for each shRNA. **(C)** Validation of the existence of miR-202-3p binding sites in the 3′UTRs of *nfkbiaa*, *perp* and *mgll* genes by dual luciferase assays in HEK29T cells. Luciferase activities from constructs containing the 3′UTRs of wild type (WT) or mutated miR-202-3p binding site (MUT) for each gene were measured comparatively. NC, scrambled miR-202-3p; Agomir, synthetic miR-202-3p. Error bars, mean ± s.d., *n* = 3 (biological replicates).

Using miRNAMap2 (Hsu et al., 2008) and TargetScanFish (Ulitsky et al., 2012), we identified 24 possible target genes of miR-202-3p from the DEGs (Supplementary Table S4). We then selected seven upregulated genes (*nfkbiaa*, *mgll*, *nfil3-5*, *atp1b1a*, *pleca*, *nfe2l2b* and *perp*) for further analysis based on their potential involvement in cell proliferation and apoptosis, cell adhesion, and metabolism (Table 1). Since all these genes are up-regulated when miR-202-3p is deleted or knocked down, we first tested whether minimizing of the elevation of these transcripts could alleviate the fatal phenotype when a miR-202-3p antagomir was present. We thus co-injected miR-202-3p antagomir with a designed shRNA targeting one of the seven genes (Figure 6A). We verified that the gene-specific shRNAs functioned properly to suppress the up-regulation of the intended genes before and after 4 hpf (Supplementary Figure S2). We counted the number of normally developing embryos for each of the shRNAs at 6 hpf–the timepoint when the miR-202-3p antagomir would have led to cytolysis if no shRNA had been injected. We found that down-regulated expression of three genes: *nfkbiaa*, *perp* and *mgll* was able to rescue about 80, 50 and 45% of the miR-202-3p knockdown embryos from developmental failures at MBT, respectively (Figure 6B).

**TABLE 1:** Screened target genes.

Gene	Description	Gene ID	GO: Biological process
*Nfkbiaa*	nuclear factor of kappa light polypeptide gene enhancer in B-cells inhibitor, alpha a	ENSDARG00000005481	Apoptosis; regulation cell differentiation.
*mgll*	monoglyceride lipase	ENSDARG00000036820	indirect involved in apoptosis
			lipid metabolic process
			regulation of signal transduction
*nfil3-5*	nuclear factor, interleukin 3 regulated, member 5	ENSDARG00000094965	Apoptosis; regulation of transcription.
*atp1b1a*	ATPase Na^+^/K^+^ transporting subunit beta 1a	ENSDARG00000013144	establishment or maintenance of transmembrane electrochemical
			gradient positive regulation of ATPase activity
			ion transport
*pleca*	plectin a	ENSDARG00000062590	skeletal muscle tissue development
			locomotory behavior
			cytoskeleton organization
*nfe2l2b*	nuclear factor, erythroid 2-like 2b	ENSDARG00000089697	positive regulation of transcription, DNA-templated
			negative regulation of transcription in response to oxidative stress
*perp*	p53 apoptosis effector related to pmp22	ENSDARG00000063572	regulation of apoptotic process

To verify that these three genes are direct targets of miR-202-3p, native and mutated forms of the 3′UTRs of these genes were cloned to a luciferase report vector (Supplementary Figure S3). Both native and mutated vector were co-transfected with miR-202-3p agomir and scrambled agomir into HEK293T cells to measure luciferase activities. Results validated that *nfkbiaa*, *perp* and *mgll* are direct targets of miR-202-3p (Figure 6C).

### *nfkbiaa*, *perp* and *mgll* are essential for early embryo development

**FIGURE 7:**
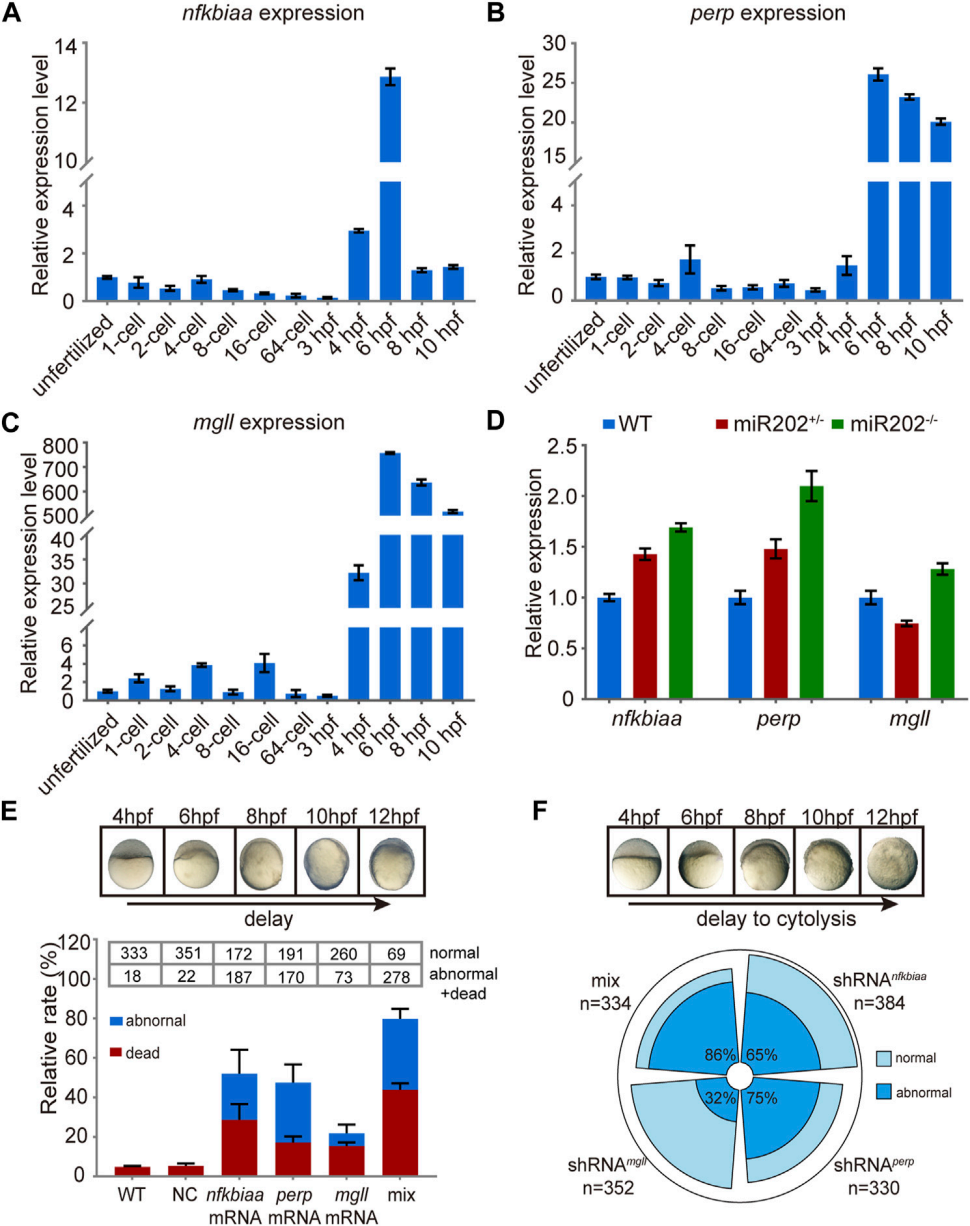
*In vivo* validation of the involvement of *nfkbiaa*, *perp* and *mgll* in zebrafish early development. **(A–C)** qRT-PCR analysis of *nfkbiaa* **(A)**, *perp* **(B)** and *mgll* **(C)** expression during the course of early embryonic development from unfertilized embryo to 10 hpf. **(D)** Quantitative analysis of homozygous, heterozygous and wild-type embryos revealed that the expression level of *nfkbiaa*, *perp* and *mgll* was overexpressed in miR-202 homozygous mutant. **(E)** Time-matched bright field images of slow-developing embryos (top) and the rate of mortality and developmental abnormality (bottom) when extra mRNA of *nfkbiaa*, *perp* and *mgll* were introduced to wild type embryos (bottom). The “mix” indicates mixture of equal amounts of mRNAs of the three genes. The statistical assays were performed using data obtained at 12 hpf. **(F)** Time-matched bright field images of embryo showing developmental delay and cytolysis (top) and the rate of developmental abnormality (bottom) when shRNA of *nfkbiaa*, *perp* and *mgll* were introduced to wild type embryos. The “mix” indicates mixture of equal amount of shRNAs of the three genes. Error bars, mean ± s.d., *n* = 3 (biological replicates).

We further investigated the inter-relationship among the trio of miR-202-3p target genes. We first examined the expression patterns of the trio in wild type embryos and in miR-202^−/−^ embryos by qRT-PCR. Data showed that all three genes were transcribed around 4 hpf during ZGA (Figures 7A–C) and were overexpressed when miR-202 is deleted (Figure 7D), coinciding the timing of the fatal phenotype of the miR-202^−/−^ mutant. To explore the relative roles of the trio in generating the phenotype, we cloned the transcripts of these genes, *in vitro* transcribed them, and microinjected them into fertilized eggs of wild type fish. Examining at 12 hpf, approximately 80%, 50%, 45% and 20% of injected embryos exhibited developmental delay or embryonic mortality when mixture of the three target gene mRNAs, *nfkbiaa* mRNA, *perp* mRNA and *mgll* mRNA were introduced, respectively (Figure 7E). These results further validated involvement of all three genes in producing the phenotype, but *nfkbiaa* and *perp* played bigger roles in the process than *mgll*. Interestingly, similar ratios of developmental delay and embryonic mortality were observed when the trio were supressed by their respective shRNAs or a mixture of the three shRNAs in wild type embryos (Figure 7F). The similar fatal phenotypes produced by over- and down-regulation of the trio indicated transcription of the trio, especially those of *nfkbiaa* and *perp*, need to be tightly controlled for proper embryogenesis to proceed. These findings suggested the need of tight control of miR-202-3p level during MBT.

### *nfkbiaa*, *perp* and *mgll* regulate early development via an inter-regulated network

**FIGURE 8:**
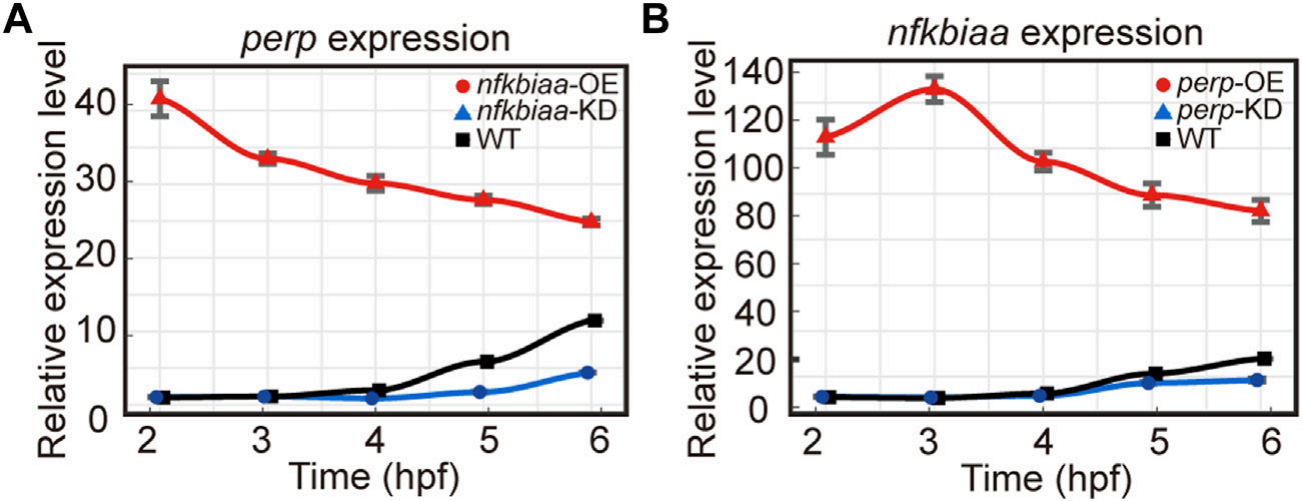
*nfkbiaa* and *perp* are inter-regulated in early embryonic development. **(A)** The mRNA expression level of *perp* response to overexpression (by mRNA microinjection) and downregulation (by shRNA microinjection) of *nfkbiaa* in zebrafish embryos observed from 2 hpf to 6 hpf. **(B)** The mRNA expression level of *nfkbiaa* response to overexpression (by mRNA microinjection) and downregulation (by shRNA microinjection) of *perp* in zebrafish embryos observed from 2 hpf to 6 hpf. Error bars, mean ± s.d., *n* = 3 (biological replicates).

As the similar phenotypes resulted from over- or down-regulation of the individual genes hinted potential existence of inter-regulation among the trio. The notion was proved true as we found that when *nfkbiaa* was over-expressed or down-regulated, transcription of *perp* responded in the same direction during the developmental period from 2-6 hpf (Figure 8A). Similarly, over-expression or down-regulation of *perp* elicited the same responses from *nfkbiaa* (Figure 8B). Therefore, *nfkbiaa* and *perp* are inter-regulated. An inter-regulation relationship between genes was also true between *nfkbiaa* and *mgll*, but in a slightly different manner, in which over- and under-expression of *nfkbiaa* resulted in down-regulation of *mgll* (Supplementary Figure S4). Our results showed that *nfkbiaa*, *perp* and *mgll* form an interconnected regulatory network and the two major factors, *nfkbiaa* and *perp* are positively regulating on each other.

**FIGURE 9:**
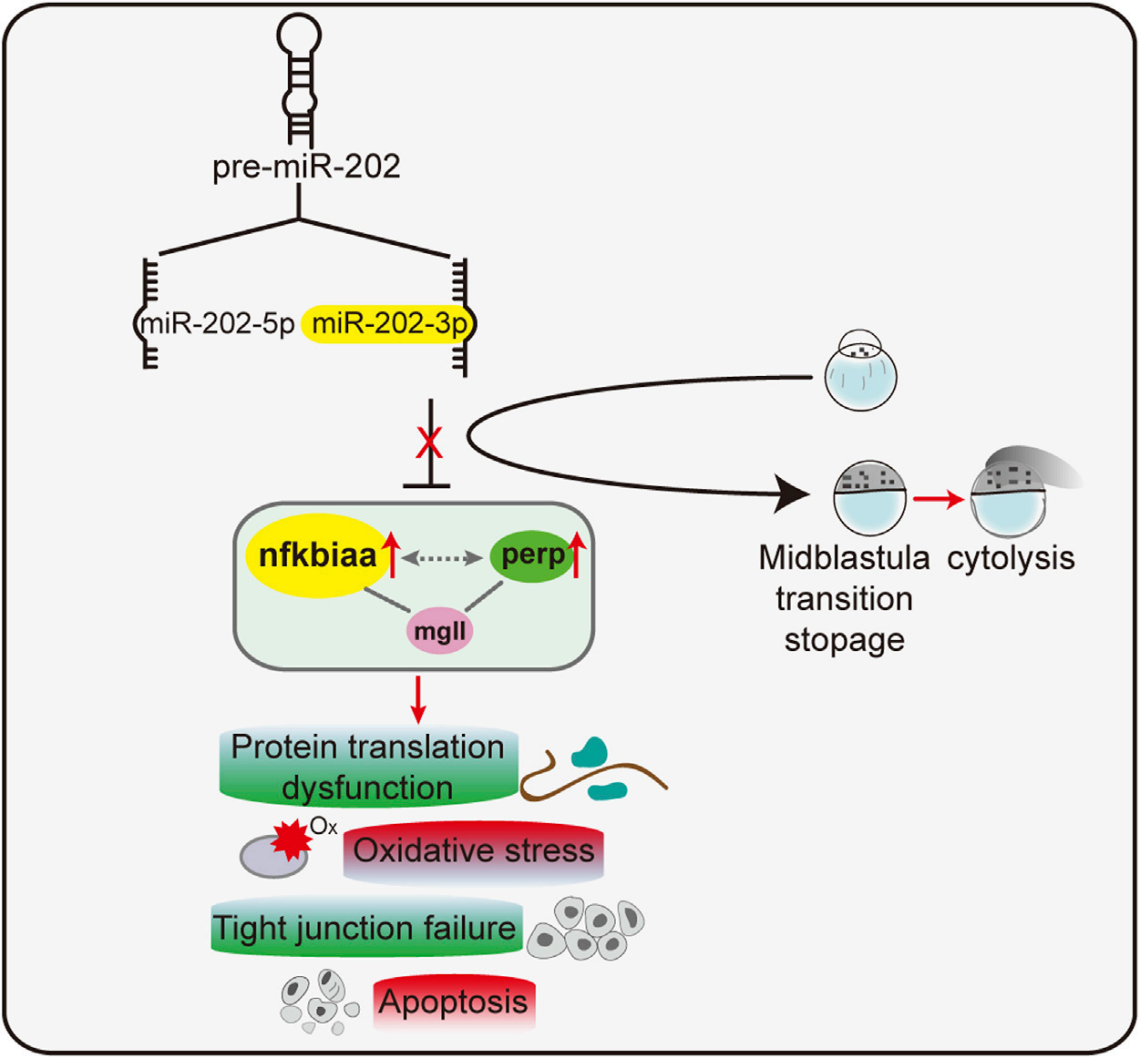
A proposed model of a miR-202-3p mediated regulatory network that determines embryonic viability during MBT in zebrafish.

Taking all results together, we concluded that miR-202-3p suppressed over-expression of *nfkbiaa*, *perp* and *mgll* during MZT. These three target genes formed interconnected regulatory networks with each other. Reciprocally, *nfkbiaa* and *perp* regulate expression of the miR-202 locus, these inter-regulation loops acted concertedly to maintain miR-202-3p at a relatively constant level (Figure 9). This tightly regulated miR-202-3p-mediated network is essential to maintain embryonic viability during MBT.

## Discussion

In this study, we accumulated solid evidence indicating that miR-202-3p plays essential roles to maintain embryonic viability during the mid-blastula transition in zebrafish. Unlike the miR-430s and miR-30 which function during MZT with tens of thousands of sequencing reads, miR-202 functions singularly with only single digits of sequencing reads (Supplementary Figure S5A) (Wei et al., 2012). miR-202 is located adjacent to *vent* and *vox* in the long arm of chromosomal 13. *Vox* and *vent* are known centralizing factors activated prior to or around the period of zygotic genome activation (Imai et al., 2001; Gilardelli et al., 2004; Pshennikova et al., 2017). Analysis of the abundance of the miR-202 products (miR -202-3p and miR -202-5p) in early development (from unfertilized cell to 10 hpf) indicated that the expression level of miR -202-5p was significantly higher than that of miR -202-3p (Supplementary Figure 5B). Although miR-202-3p normally is present only in a low concentration, the knock down or deletion of miR-202-3p resulted in the drastic fatal phenotypes at MBT. Especially convinced by the fatal phenotypes of the miR-202 null embryos are rescued only by miR-202-3p or its precursor and not by miR-202-5p. These findings indicate that miR-202-3p is low in expression but essential for embryonic survival during MBT. It is so far the only miRNA found functioning in the initial stages of embryogenesis with a life/death effect.

miR-202-3p plays an essential role in MZT adds to the list of known players of this important developmental transition, such as miR-430s, Ythdf2, nanog, pou5f3 and others (Lee et al., 2013; Pálfy et al., 2020). Unlike mutations of the other known factors that usually impair cell differentiation or lineage commitment during embryogenesis, loss of miR-202-3p resulted in catastrophic breakdown of cellular homeostasis, which eventually leads to massive apoptosis and cytolysis of the blastomere. We found distinctive pathways among the three subgroups of dysregulated genes of miR-202 null embryos. Specifically, genes showing insufficient degradation included genes belonging to the categories “Ribosome” and “oxidative phosphorylation.” Over-expressed genes included those belonging to the categories “Tight junction”, “Apoptosis” and a few pathways related with inflammation. While a few pathways of biosynthesis and metabolism were represented by the insufficiently expressed genes. Correspondingly, we were able to verify such homeostatic dysfunctions as slowed protein synthesis, elevated oxidative stress, loss of tight junctions and apoptosis in the miR-202^−/−^ embryos. Remarkably, the presence of 7 less degraded ribosomal protein mRNAs did not translate into higher levels of these ribosomal proteins; instead many of the ribosomal proteins were significantly reduced and the rate of protein synthesis was impaired. How the hindered clearance of the ribosomal protein mRNAs influenced ribosome biogenesis needs to be further explored. Successful rescue of the fatal phenotypes by miR-202-3p suggested that much of the basis of these phenotypes might be attributable to the dysregulation of the miR-202-3p target genes in the miR-202 null embryos.

By a target screen followed with *in vivo* verification, *nfkbiaa*, *perp* and *mgll* were identified as direct targets of miR-202-3p. *nfkbiaa* is the fish homolog of mammalian *NFκBIA*, and its protein product is IκBa. It has been reported that NFκB activation of target gene expression could be one of the first events in a cascade leading to major embryo genome activation (EGA) (Halstead et al., 2020). In *Xenopus*, NF-κB activation is observed during oocyte maturation (Dominguez et al., 1993) and in late blastulae and gastrulae (Richardson et al., 1994). In mouse embryos, activation of NF-κB is required for the development of mouse embryos beyond the 2-cell stage (Nishikimi et al., 1999). In human, epididymal embryonic development harbors NFκB signaling pathway as a morphogenetic player (Ferreira et al., 2022). Both in the mouse and human, NF-κB as a critical regulator of fertility is associated with oxidative damage via activation of NF-κB (Tatone et al., 2008). Another miR-202-3p target gene identified was *perp*. *perp* contains multiple binding motifs for the tumorous suppressor protein P53 in the promoter region, and transcription of *perp* is directly activated by P53 (Attardi et al., 2000). *perp* is a mediator of *p53*-dependent apoptosis in diverse cell types (Qian et al., 2020; Yuan et al., 2020). Nowak et al. (Nowak et al., 2005) reported that one-to two-cell stage zebrafish embryos injected with *in vitro* synthesized *perp* mRNA displayed a severely malformed body shape at 24 hpf due to enhanced cell death during gastrulation and segmentation stages. The third direct target of miR-202-3p is *mgll*. Some studies show that MGLL is a key enzyme in the lipid metabolism network by supplying free fatty acids for β-oxidation and for providing components to build cell structures and effector molecules which are involved in cell proliferation, invasion, apoptosis resistance and stemness (Nomura et al., 2011; Das et al., 2013; Sun et al., 2013; Zhang et al., 2016). Overexpression of MGLL suppressed cell migration and induced cell death that was coupled with caspase activation (Yang et al., 2018; Liu et al., 2020). In this study, we showed deletion of miR-202 (and thus miR-202-3p) resulted in an elevated *nfkbiaa*, *perp* and *mgll* mRNA in the blastula. The detrimental effect of over-expressed the trio genes were rescued to a substantial degree by its inhibitory shRNA, indicated that a certain level of target gene is essential for cell viability during MBT in zebrafish.

Multiple studies in cancerous cells have shown that *nfkbiaa* and *perp* are inter-regulated. It has been reported that *perp* expression stabilizes active *p53* via modulation of p53-MDM2 interaction, thus forming a positive feedback between *perp* and *p53* activity (Davies et al., 2011). On the other hand, IκBα is an interacting partner of P53 and formation of the p53/IκBα complex generally has an inhibitory effect on *p53* activity (Carrà et al., 2016). Thus, *nfkbiaa* and *perp* are interconnected through pathways involving *p53*. In this study, we found that knockdown of either one led to reduced expression of the other, and over-expression of either one caused elevated transcription in the other, suggesting a positive inter-regulation between the two. Whether the inter-regulation between *nfkbiaa* and *perp* could be mediated by the P53 signaling pathway in zebrafish embryos warrants further investigation.

Many universal key post-transcriptional mechanisms are known to contribute to maternal mRNA clearance. MicroRNA-dependent mechanisms, which often promote deadenylation is common, with the miR-430 destabilizing hundreds of mRNAs in zebrafish (Bazzini et al., 2012), miR-427 acting in *Xenopus* (Lund et al., 2009), and miR-309 functioning in *Drosophila* (Bushati et al., 2008). In addition, Ythdf2 N6-methylation (m6A) also drives mRNA deadenylation of maternally provided mRNAs, whose decay is essential for zebrafish embryogenesis (Kontur et al., 2020), mouse embryonic stem cells development (Geula et al., 2015)and murine oogenesis (Ivanova et al., 2017). The targets of miR-430 and Ythdf2 exhibit notable overlap, and their common target decay earliest, followed by the specific targets of Ythdf2, and then the specific targets of miR-430 (Zhao et al., 2017). These factors suggest the importance of timely, robust removal of maternal transcripts by overlapping yet temporally distinct mechanisms for proper development (Stoeckius et al., 2014). MicroRNA-30a regulates zebrafish myogenesis through targeting the transcription factor Six1 (O'Brien et al., 2014). In our study, miR-202-3p targets the *nfkbiaa*, *perp* and *mgll* to regulate zebrafish embryonic development. Therefore, whether miR-202-3p or miR-30a has the function of deadenylation is still unknown and the specific mechanism needs to be further explored.

To summarize, we showed that miR-202-3p is essential to maintain cellular viability during MZT through modulating maternal mRNA degradation and zygotic transcription. We identified a trio of genes, *nfkbiaa*, *perp* and *mgll* that are direct targets of miR-202-3p and are inter-regulated by each other. Dysregulation of the regulatory network is directly linked to slower development progression and apoptosis through influencing the rate of protein synthesis, ROS clearance, cell adhesions and apoptosis during MBT.

## Data Availability

The datasets presented in this study can be found in online repositories. The names of the repository/repositories and accession number(s) can be found below: NCBI, accession no. PRJNA529372.
