# Supplementary material for: MiR-202-3p determines embryo viability during mid-blastula transition
Source: Front Cell Dev Biol. 2022 Aug 8;10:897826. doi: 10.3389/fcell.2022.897826 (PMC9393261; doi:10.3389/fcell.2022.897826)
Supplement: Supplementary file 6 [file datasheet1.docx]

**Supplementary Figures and Legends**


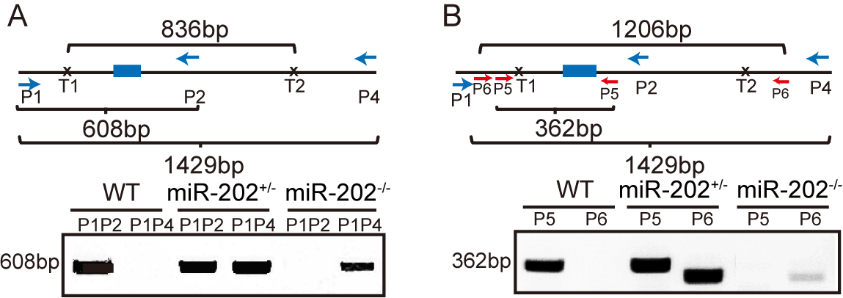


Supplementary Figure S1 **Genotype identification of miR-202 mutant (miR-202^+/-^ x miR-202^+/-^ ).** (A) Illustration of genotyping strategy. PCR was performed simultaneously using two sets (P1P2 and P1P4) of primers on genomic DNAs extracted from fish tails or embryos, and genotypes were determined according to the size of the PCR products after electrophoresis (bottom). (B) The scheme of nested PCR for genotyping of embryos prior to 10 hpf. PCR was performed simultaneously using two sets (P5 and P6) of primers on PCR product from P1P2 (for P5) and P1P4 (for P6), and genotypes were determined according to the size of the PCR products after electrophoresis (bottom).


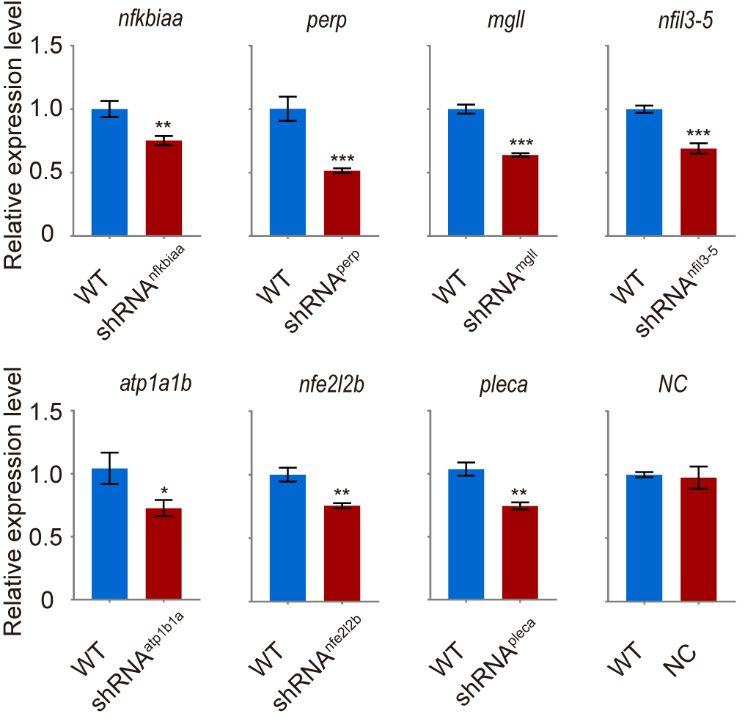


Supplementary Figure S2 **Validation of the suppression efficiency of the shRNAs targeted to the selected genes.** Each shRNA was microinjected into wild type embryos at the 1-cell stage at a concentration of 200 ng/µl. Empty plasmid vector was used as negative control (NC). Error bars, mean± s.d., n= 3 (biological replicates).


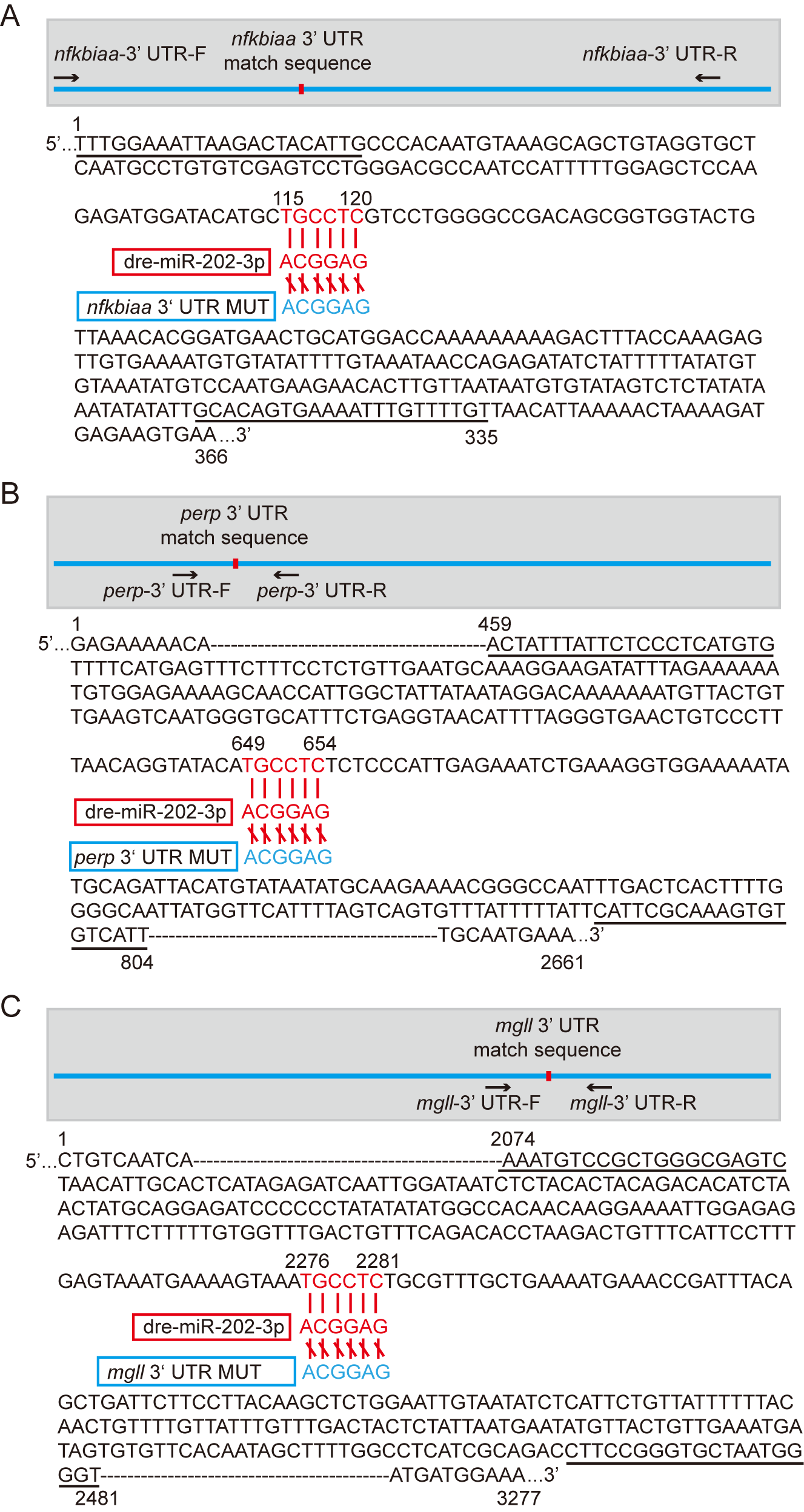


Supplementary Figure S3 **Native and mutated sequence of the 3’UTRs were used in dual luciferase assays to verify that *nfkbiaa*, *perp* and *mgll* are direct targets of miR-202-3p.** The native 3’UTRs that can match with miR-202-3p seed sequence are in red, and the mutant 3’UTRs (MUT) that mismatch with the miR-202-3p seed sequence are in blue. Primer sequences used to clone a portion of 3’UTR are underlined.


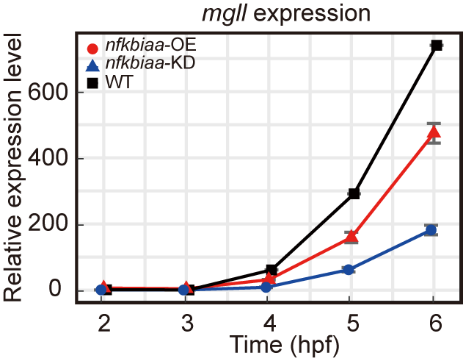


Supplementary Figure S4 **Expression of *mgll* is influenced by over- or down- expression of *nfkbiaa* in developing zebrafish embryos measured at different time intervals (hpf).** Error bars, mean± s.d., n= 3 (biological replicates).


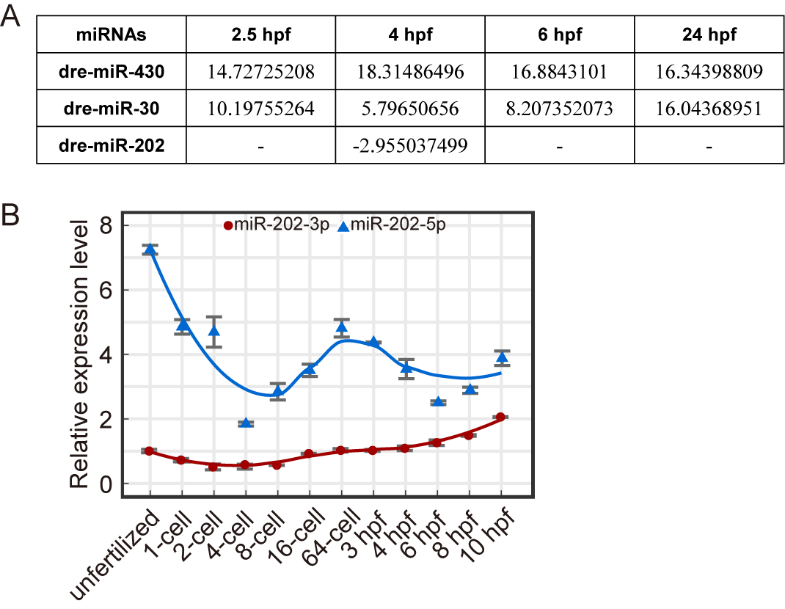


Supplementary Figure S5 The expression patterns of miR-430, miR-30 and miR-202 during early embryonic development. (A) Transcriptome-wide analysis of small RNA expression in early zebrafish embryo development (2.5 hpf, 4 hpf, 6 hpf, and 24 hpf). Normalized expression levels of miRNA families were indicated as log2 value of the reads number of miRNA families across 4 development stages (Wei et al., 2012). (B) qRT-PCR analyses of the dynamic expression of miR-202-3p (in red) miR-202-5p (in blue) through early developmental time course (from unfertilized cell to 10 hpf). Error bars, mean± s.d., n= 3 (biological replicates).

**Supplementary Tables**

Supplementary Table S1. DEGs of RNA_seq

Supplementary Table S2. Genes of different KEGG pathway

Supplementary Table S3. Protein_seq and ribsome related proteins

Supplementary Table S4. Predicted targets of miR-202-3p

Supplementary Table S5. List of various sequences used in the experiment
